# Continuous glucose monitoring for diabetes prevention in adults with prediabetes: a scoping review

**DOI:** 10.3389/fendo.2026.1965925

**Published:** 2026-10-28

**Authors:** Hye Jung Lee, Jin Kyeong Kim

**Affiliations:** Department of Internal Medicine, Dong-A University Hospital, Busan, Republic of Korea

**Keywords:** continuous glucose monitoring, diabetes prevention, glycemic variability, prediabetes, scoping review, time in range

## Abstract

**Background:**

Prediabetes affects a rapidly growing proportion of adults worldwide (IDF Diabetes Atlas 2024: 12.0% impaired glucose tolerance; 9.2% impaired fasting glucose). Continuous glucose monitoring (CGM) offers a more granular window into glycemic patterns than intermittent testing, and its use in prediabetes research has grown substantially, but this literature has not been comprehensively mapped.

**Objective:**

To systematically map the extent, range, and nature of peer-reviewed literature on CGM use in adults with prediabetes, following a pre-registered protocol.

**Methods:**

PubMed was searched in June 2026 (436 records), followed by a supplementary Europe PMC and Scopus check in October 2026. After AI-assisted first-pass title/abstract screening (with *post hoc* human re-screening of random samples) and independent two-reviewer eligibility assessment against Population–Concept–Context criteria (adults 18 and older with prediabetes; any rtCGM/isCGM use; no design restriction), 44 PubMed studies were included. The supplementary search added 4, a *post hoc* re-screening audit reinstated 1, and 1 was excluded on closer verification, giving 48 included studies, all with extracted data. No meta-analysis or formal quality appraisal was performed; studies were narratively synthesized into five pre-specified categories.

**Results:**

Fifteen studies characterized CGM-derived glycemic metrics, showing graded worsening of glycemic control and variability across the normoglycemia–prediabetes–diabetes continuum. Twenty-nine studies evaluated CGM-guided interventions, which produced CGM-detectable improvements in glycemic variability or time-in-range that did not consistently translate into significant HbA1c or fasting glucose change. Three studies addressed screening/diagnosis, including a large cohort (n=8,315) in which single-timepoint fasting glucose resulted in different glycemic classification when repeated CGM-derived measurements were considered. One study addressed broader metabolic/complication risk, and none addressed CGM-based prediction of progression from prediabetes to type 2 diabetes.

**Conclusion:**

CGM provides a more granular, continuous picture of glycemic dysregulation in prediabetes than intermittent testing. Interventions used CGM either as a real-time feedback tool or as a blinded outcome measure of non-CGM interventions, with CGM-detectable glycemic improvement in both, though durable biomarker translation remains inconsistent. CGM-based prediction of progression to diabetes is an unaddressed evidence gap; CGM’s role in screening/diagnosis and complication-risk assessment also warrants more research.

**Systematic review registration:**

https://doi.org/10.17605/OSF.IO/QSXK9, identifier 10.17605/OSF.IO/QSXK9.

## 1 Introduction

Prediabetes, an intermediate state of hyperglycemia that falls short of a diabetes diagnosis but confers substantially elevated risk of progression to type 2 diabetes, has become a global public health concern of considerable scale. Based on International Diabetes Federation (IDF) Diabetes Atlas data, updated 2024 estimates place the global prevalence of impaired glucose tolerance (IGT) at 12.0% and impaired fasting glucose (IFG) at 9.2% of the adult population, both figures having risen substantially from 2021 estimates (9.1% and 5.8%, respectively), with 487.7 million adults affected by IFG alone (1, 2). Adults with prediabetes are at markedly increased risk not only of progressing to overt type 2 diabetes but also of cardiovascular disease and other cardiometabolic complications (3), making early identification and effective intervention essential priorities in clinical care and public health.

Beyond the direct effects on health, this progression also carries a large economic cost. In the United States, diagnosed diabetes cost an estimated $412.9 billion in 2022, including $306.6 billion in direct medical expenditures, with people living with diabetes incurring average annual excess health care costs of $12,022 per person compared with those without diabetes (4), whereas Stelo, the first FDA-cleared over-the-counter CGM marketed specifically for adults with prediabetes and non-insulin-treated type 2 diabetes, costs approximately $89 to $99 per month, or roughly $1,100 per year, without a prescription (5). If CGM-derived metrics could help identify which individuals with prediabetes are most likely to progress, this could enable risk-stratified targeting of intensive prevention programs, such as the Diabetes Prevention Program (DPP), toward those most likely to benefit. Thus, whether CGM can, in practice, help reduce progression from prediabetes to type 2 diabetes is the central question this review sought to address.

Current diagnostic and monitoring approaches for prediabetes rely on single-timepoint or intermittent measures: fasting plasma glucose, the 2-hour oral glucose tolerance test (OGTT), and glycated hemoglobin (HbA1c) (6). Each of these tests provides only a snapshot of glycemic status. Considerable evidence, some of it summarized in this review, indicates that day-to-day and within-day glycemic variability in this population can be substantial, raising the possibility that intermittent testing may under- or over-classify an individual’s true glycemic status.

Continuous glucose monitoring (CGM), including both real-time CGM (rtCGM) and intermittently scanned/flash CGM (isCGM), offers a fundamentally different window into glycemic control, generating dense, longitudinal glucose data over days to weeks. CGM was first developed and validated for use in diabetes management, where its accuracy has improved substantially over the past decade (7). It is now supported by international consensus guidelines defining standardized metrics, such as time in range and glycemic variability indices, and target thresholds for clinical and research use (7). Uptake of CGM has since expanded well beyond insulin-treated diabetes, including growing research interest in its application to prediabetes. This includes use as a tool to better characterize glycemic patterns in this population, and as a potential feedback mechanism to support behavioral interventions aimed at diabetes prevention. Such interventions build on evidence from the original Diabetes Prevention Program (DPP) (8).

Despite this growing research interest, the literature on CGM in prediabetes remains fragmented across widely differing study designs, populations, CGM metrics, and research aims, ranging from small mechanistic studies of glycemic variability to large digital-health intervention trials to methodological or diagnostic validation studies. A recent Viewpoint article by Black and colleagues (9) took an important step toward addressing this fragmentation. It characterized the methodological features, including study design, sensor wear protocols, masking strategies, and CGM metric reporting, of 19 peer-reviewed studies using CGM specifically within behavioral interventions in prediabetes, identified through a pragmatic, non-systematic literature review. That work provides valuable methodological guidance for behavioral-intervention researchers. However, by design it does not address CGM’s use for glycemic characterization, screening/diagnosis, or complication-risk assessment in prediabetes. It also does not employ a systematic search and screening methodology. As a result, the true scope of the broader literature, including the evidence gaps within it, has not yet been comprehensively mapped.

To our knowledge, no prior systematic or scoping review has comprehensively mapped the full breadth of CGM use in adults with prediabetes. Existing work has tended to focus on a single sub-application rather than the full range of research purposes, including characterization, intervention, screening/diagnosis, progression prediction, and complication-risk assessment. This scoping review was therefore designed to address that gap.

### 1.1 Objectives

The objective of this scoping review was to systematically identify and map the existing peer-reviewed literature on the use of CGM in adults with prediabetes, following the Population–Concept–Context (PCC) framework: adults aged 18 years or older with prediabetes (Population), any use of rtCGM or isCGM in any research capacity (Concept), with no restriction on study design or context (Context). Consistent with scoping review methodology, this review aims to describe the extent, range, and nature of this literature rather than to pool effect sizes or appraise study quality, and to identify areas where the evidence base is well developed versus areas warranting further primary research. Findings are organized into five categories that were pre-specified in the registered protocol: 1) CGM metrics characterization in prediabetes; 2) CGM-guided interventions; 3) CGM for detection/screening; 4) CGM and progression to diabetes; and 5) CGM and metabolic/complication risk.

## 2 Methods

This scoping review was conducted and is reported in accordance with the Preferred Reporting Items for Systematic Reviews and Meta-Analyses extension for Scoping Reviews (PRISMA-ScR) checklist.

### 2.1 Protocol and Registration

The protocol for this scoping review was prospectively registered on the Open Science Framework (OSF) prior to the commencement of screening (Registration DOI: 10.17605/OSF.IO/QSXK9). Eligibility criterion EC5 had been part of the originally planned eligibility criteria but was inadvertently omitted from the initial OSF registration text; on review of the registered protocol, this omission was identified and corrected via a protocol amendment filed before screening began, such that all screening and full-text review described below (Sections 2.2–2.4) was conducted with EC1–EC5 in place from the outset. This amendment did not represent a substantive change to the eligibility criteria as originally planned, since EC5 reflected the review’s originally intended scope rather than a newly introduced criterion.

### 2.2 Eligibility criteria

Eligibility was defined using the Population–Concept–Context (PCC) framework recommended for scoping reviews.

**Population:** Adults aged 18 years or older with prediabetes, defined per American Diabetes Association (ADA) criteria (HbA1c 5.7–6.4%, impaired fasting glucose [IFG], or impaired glucose tolerance [IGT]) or World Health Organization (WHO) criteria (IFG or IGT) (10).

**Concept:** Any use of real-time CGM (rtCGM) or intermittently scanned/flash CGM (isCGM), in any capacity (e.g., as a primary or secondary outcome measure, an intervention/feedback tool, or a descriptive/characterization tool).

**Context:** No restriction was placed on study design, setting, or geographic location. Both interventional and observational studies were eligible.

Studies were excluded during full-text review if they met any of the following pre-specified eligibility criteria (EC), as defined in the registered protocol:

EC1: Established type 1 or type 2 diabetes only, with no prediabetes subgroup.
EC2: Pediatric/adolescent population (age <18 years).
EC3: Review article, editorial, case report, or case series (i.e., not a primary research report); preprints not published in a peer-reviewed journal and study protocols without results were likewise treated as not primary research reports.
EC4: Non-human (animal or *in vitro*) studies.
EC5: CGM was used solely as a secondary measurement tool, with a primary outcome unrelated to glycemic control or diabetes prevention (e.g., cardiovascular events, autonomic neuropathy, or acute kidney injury as the primary endpoint, with CGM used to characterize the study population rather than to evaluate glycemic outcomes).

Of the 30 records excluded at eligibility assessment, EC1 accounted for 9, EC3 for 7, and EC5 for 14; no records were excluded under EC2 or EC4, as none of the 89 records assessed involved a pediatric or non-human population at this stage. Studies published only in a language other than English, for which no English-language full text was available, were also excluded and are reported separately in the Results as a language-related exclusion, distinct from EC1–EC5.

### 2.3 Information sources and search strategy

A single electronic database, PubMed, was searched on June 29, 2026 (the date of OSF protocol registration), using the Boolean search string registered in the study protocol (Registration DOI: 10.17605/OSF.IO/QSXK9): “(prediabetes[MeSH Terms] OR prediabetes[tiab] OR “pre-diabetes”[tiab] OR “impaired glucose tolerance”[tiab] OR “impaired fasting glucose”[tiab] OR “intermediate hyperglycemia”[tiab] OR “dysglycemia”[tiab]) AND (“continuous glucose monitoring”[tiab] OR “continuous glucose monitor”[tiab] OR CGM[tiab] OR “flash glucose monitoring”[tiab] OR “intermittently scanned CGM”[tiab] OR isCGM[tiab] OR FGM[tiab] OR “FreeStyle Libre”[tiab] OR “Dexcom”[tiab])”. No date or language restriction was applied at the search stage, and no species (e.g., “Humans”) filter was applied; non-human and *in vitro* records were instead excluded during screening under EC4 (Section 2.2). The single-database search strategy is acknowledged as a limitation of this review; to partly address this, supplementary searches of Europe PMC and Scopus were performed on October 2, 2026 using queries adapted from the registered PubMed string (full query text in the **Supplementary File 1**: Multi-Database Literature Search Documentation; record-level results in **Supplementary Table S1**). The flow of the supplementary search is shown in **Figure 1**, and its results are reported in the Discussion (Section 4.5, Limitations); records not in the PubMed export were screened on title/abstract with AI assistance (full text reviewed where needed), and a random sample of 100 of the AI-excluded records was re-screened by two authors (Section 4.5). The PubMed search yielded 436 records.

**Figure 1:**
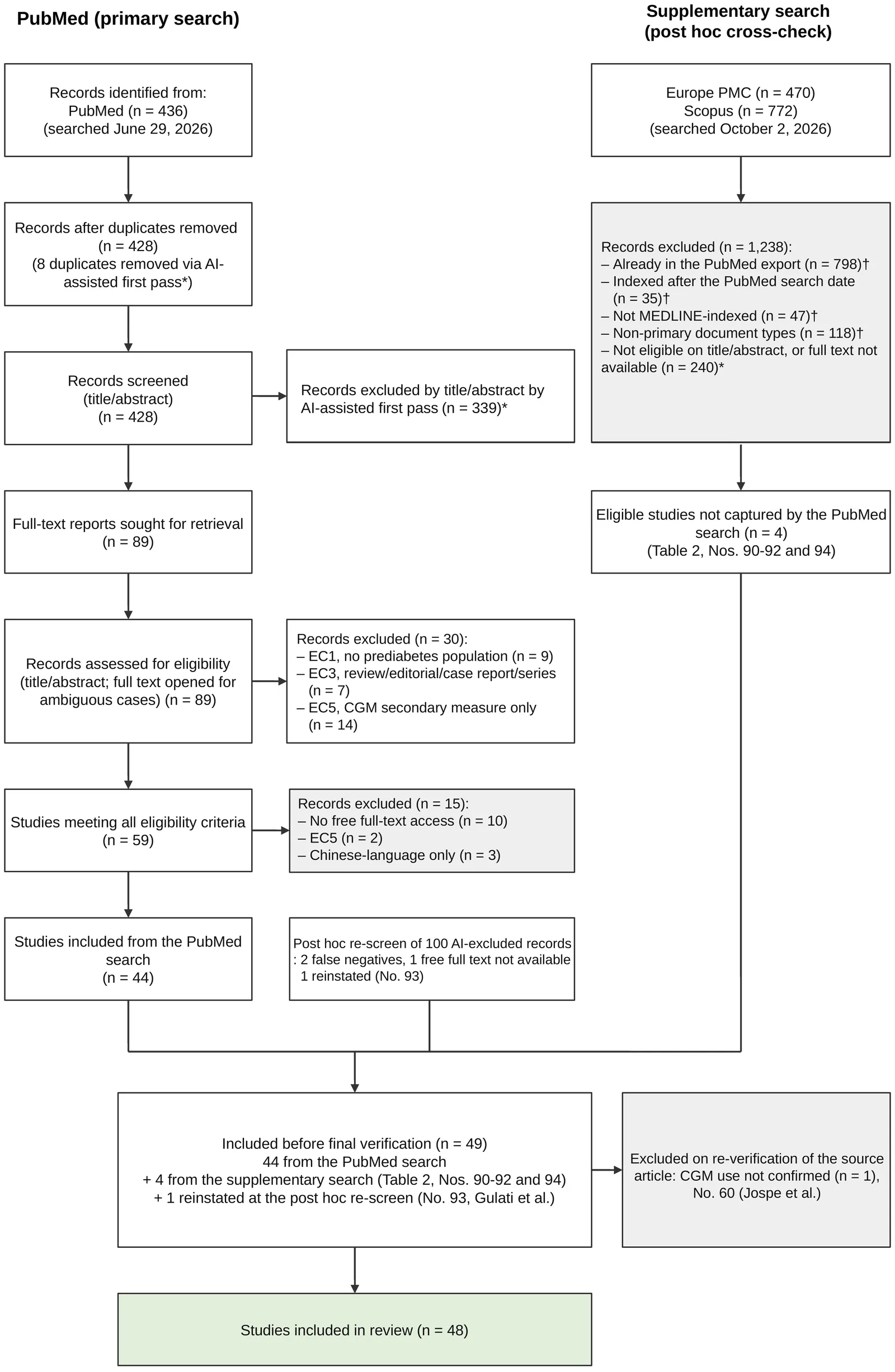
PRISMA-ScR flow diagram. Of the 59 records provisionally included after eligibility review (title/abstract, with full text opened for ambiguous cases), 10 had no freely accessible full text (via PubMed Central [PMC] or the publisher’s own open-access site) and were excluded, as eligibility could not be fully confirmed and data could not be extracted; a further 2 were excluded under EC5 and 3 for having no English-language full text, both apparent only on closer full-text reading. This yielded a final total of 44 included studies, all with extracted data. EC1-EC5 refer to the eligibility criteria defined in Methods, Section 2.2 (only EC1, EC3, and EC5 resulted in exclusions at the eligibility stage). The supplementary search of Europe PMC and Scopus was performed on October 2, 2026 as a *post hoc* cross-check, using queries adapted from the registered PubMed string (**Supplementary File 1**: Multi-Database Literature Search Documentation; record-level results in **Supplementary Table S1**); records not in the PubMed export were screened on title/abstract by an AI-assisted pass, with full text reviewed where needed; a random sample of 100 AI-excluded records from this search was re-screened by two reviewers and contained no false negatives (0/100). The *post hoc* re-screen of the PubMed search was performed on a random sample of 100 records excluded in the AI-assisted first pass (Methods, Section 2.4; Discussion, Section 4.5). Studies from the PubMed search (n = 44), the supplementary search (n = 4) and the *post hoc* re-screen (n = 1) were combined (n = 49), and one study (No. 60) was excluded after verification of the source article, giving 48 included studies.*AI-assisted title/abstract screening (Claude Sonnet 5); not individually verified by human reviewers. Random samples of 100 AI-excluded records from the PubMed search (2 false negatives) and from the supplementary search (0 false negatives) were re-screened by two reviewers (Methods, Sections 2.3-2.4). †Rule-based exclusion by database metadata (duplicate, indexing date, indexing status, document type); no screening judgment involved.

### 2.4 Study selection

Records retrieved from PubMed (n=436) were screened in a single AI-assisted first pass: the full set of records was reviewed by a large language model (Claude Sonnet 5, Anthropic PBC), to identify duplicate records and to flag records that appeared, based on title and abstract, to meet one of the pre-specified exclusion criteria (EC1–EC5). The authors specified the eligibility framework and the exclusion criteria applied by the model; however, the model’s exclusion decisions for individual records were not confirmed record by record by the authors at the time of screening. This first pass identified 8 duplicate records and 339 records for exclusion at the title/abstract stage, yielding 89 unique records for full-text review. To estimate the accuracy of this step after the fact, two authors (H.J.L. and J.K.K.) independently re-screened a random sample of 100 records from the AI-excluded pool (fixed random seed) against the same criteria; two records were identified as false-negative exclusions (2/100; see Discussion, Section 4.5; record-level results of this and the supplementary-search sample in **Supplementary Table S2**). The remaining AI-excluded records were not individually re-screened, and the first-pass decisions were therefore not human-verified in their entirety at the time of screening, which is a limitation of this review (Discussion, Section 4.5).

Title and abstract review of these 89 records (**Supplementary Table S3**) was then conducted independently by two reviewers (H.J.L. and J.K.K.), applying the same PCC eligibility criteria and EC1–EC5 exclusion criteria described above; records that could not be confidently included or excluded on the basis of title and abstract alone were further assessed by opening the full text. Discrepancies between the two reviewers were resolved by discussion and consensus. Of 89 records, 30 were excluded (EC1, n=9; EC3, n=7; EC5, n=14), yielding 59 records that met all eligibility criteria and were provisionally included.

During the subsequent full-text review and data extraction stage, closer review of the 59 provisionally included records identified 15 records that were excluded from the final included set. Ten records had no freely accessible full text (via PubMed Central [PMC] or the publisher’s own open-access site) and were not accessible without direct purchase or a reprint request to the authors; because eligibility could not be fully confirmed and data could not be extracted for these records, they were excluded rather than retained without extracted data. Of the 49 records (59 minus the 10 without free full text) for which free full-text access was available, 2 records met EC5 on closer reading (CGM data were used exclusively to validate a statistical or machine-learning classification method, with no clinically oriented glycemic or diabetes-prevention outcome), and 3 records were available only in Chinese-language full text with no English translation, precluding data extraction under this review’s English-language screening approach. These 15 records were reclassified from provisionally included to excluded, yielding a final total of 44 included studies, all of which underwent full data extraction. This refinement applied EC5 as registered following the pre-screening protocol amendment described in Methods, Section 2.1, and did not require any further protocol amendment; it reflects the resolution of borderline cases and access limitations only fully apparent at the full-text stage. The complete study selection process is summarized in the PRISMA-ScR flow diagram (**Figure 1**).

### 2.5 Data extraction

A standardized data extraction form was developed and piloted prior to full extraction, covering 34 data items across 8 domains: 1) basic study identification (PMID, first author, year, journal, country); 2) study design; 3) participant characteristics (total and prediabetes-specific sample size, age, BMI, sex distribution, prediabetes diagnostic criteria used); 4) CGM methodology (device type, sensor model, wear duration, blinding status); 5) intervention/exposure and comparator, where applicable; 6) follow-up duration and primary outcome definition and result; 7) CGM-derived glycemic metrics (mean glucose, time in range [TIR], time above range [TAR], time below range [TBR], glycemic variability indices, glucose management indicator [GMI], and other CGM-derived metrics as reported); and 8) conventional glycemic biomarkers (HbA1c, fasting plasma glucose, and oral glucose tolerance test 2-hour glucose) where reported.

Data extraction from the full text of each included study was performed with the assistance of a large language model (Claude Sonnet 5, Anthropic PBC), under the direct supervision, verification, and final authority of the corresponding author (J.K.K.), who reviewed and approved all extracted data prior to synthesis; full disclosure of generative AI use is provided in the Acknowledgments section per journal policy. For every included study, the PMID recorded in the extraction database was independently re-verified against the study’s title, author list, and digital object identifier to guard against misattribution. Where a data item was not reported in the available full text, it was recorded as “not reported” (NR) rather than estimated or imputed; no data values were inferred or fabricated. All 48 included studies were accessed via a free full-text link (either PubMed Central [PMC] or the publisher’s own open-access site), rather than through institutional login credentials, so access did not depend on institutional subscription. Ten additional records that met eligibility criteria based on title and abstract had no such free full-text link available and were not accessible without direct purchase or a reprint request to the authors; as described in Methods, Section 2.4, these records were excluded from the final included set because eligibility could not be fully confirmed and data could not be extracted, which is acknowledged as a limitation (see Discussion).

### 2.6 Data synthesis

Given the anticipated heterogeneity in study designs, populations, and outcome metrics characteristic of scoping reviews, no meta-analysis or formal risk-of-bias/quality appraisal was performed, consistent with standard scoping review methodology; per the PRISMA-ScR guidance underlying this review’s reporting (11), critical appraisal of individual sources of evidence is not a required component of a scoping review, as scoping reviews aim to map the breadth of available evidence rather than to synthesize or grade its strength. This differs from the risk-of-bias assessment expected for systematic reviews. Findings from the 48 studies with extracted data were synthesized narratively and organized into five categories that were pre-specified in the registered protocol: 1) CGM metrics characterization in prediabetes; 2) CGM-guided interventions; 3) CGM for detection/screening; 4) CGM and progression to diabetes; and 5) CGM and metabolic/complication risk. Each included study was assigned to the single category best matching its primary research question and outcome focus by the corresponding author (J.K.K.). Descriptive counts of the included studies by synthesis category, publication year, region, design, CGM system, monitoring duration, and sample size are presented in **Table 1**; no quantitative pooling was undertaken.

**Table 1:** Descriptive quantitative summary of the included studies (n=48).

Characteristic	Category 1 (n=15)	Category 2 (n=29)	Categories 3 and 5 (n=4)	All studies (n=48)
Publication year				
2012–2018	2 (13%)	4 (14%)	0	6 (12%)
2019–2022	1 (7%)	7 (24%)	2 (50%)	10 (21%)
2023–2024	2 (13%)	7 (24%)	2 (50%)	11 (23%)
2025–2026	10 (67%)	11 (38%)	0	21 (44%)
Region of study conduct				
North America	7 (47%)	10 (34%)	1 (25%)	18 (38%)
Europe	5 (33%)	7 (24%)	1 (25%)	13 (27%)
East Asia	1 (7%)	9 (31%)	0	10 (21%)
South/Southeast Asia	2 (13%)	2 (7%)	1 (25%)	5 (10%)
Middle East	0	0	1 (25%)	1 (2%)
Oceania	0	1 (3%)	0	1 (2%)
Study design				
Randomized trial (parallel or crossover)	0	17 (59%)	0	17 (35%)
Secondary or *post hoc* analysis of an RCT	0	3 (10%)	1 (25%)	4 (8%)
Non-randomized interventional (single-arm, pre-post, sequential/non-randomized crossover)	0	7 (24%)	1 (25%)	8 (17%)
Observational (cross-sectional or cohort)	13 (87%)	2 (7%)	1 (25%)	16 (33%)
Pooled-dataset or model-development study	2 (13%)	0	1 (25%)	3 (6%)
CGM system (manufacturer, as reported)				
Abbott FreeStyle Libre	5 (33%)	14 (48%)	1 (25%)	20 (42%)
Medtronic	2 (13%)	7 (24%)	2 (50%)	11 (23%)
Dexcom	6 (40%)	3 (10%)	0	9 (19%)
Other	0	2 (7%)	0	2 (4%)
Not reported	2 (13%)	3 (10%)	1 (25%)	6 (12%)
CGM wear duration per monitoring period				
≤3 d	2 (13%)	9 (31%)	0	11 (23%)
4–7 d	3 (20%)	4 (14%)	3 (75%)	10 (21%)
8–14 d	9 (60%)	15 (52%)	1 (25%)	25 (52%)
>14 d	1 (7%)	1 (3%)	0	2 (4%)
Prediabetes sample size				
Median (range) of prediabetes participants per study	59 (14–463)	33 (6–253)	109 (10–2,718)	45 (6–2,718)
Intervention or follow-up duration per condition (Category 2 only)				
≤1 wk	–	9 (31%)	–	9 (31%)
>1–13 wk	–	12 (41%)	–	12 (41%)
>13–26 wk	–	6 (21%)	–	6 (21%)
>26 wk	–	2 (7%)	–	2 (7%)

Narrative synthesis categories: Category 1, CGM metrics characterization in prediabetes; Category 2, CGM-guided interventions; Category 3, CGM for detection/screening; Category 5, CGM and metabolic/complication risk. No study was classified in Category 4 (CGM and progression to diabetes). Categories 3 and 5 are combined because of small numbers. Values are n (column %), except sample size (median, range); percentages may not sum to 100 because of rounding. Counts are of studies, not participants; companion reports from the same trial (Nos. 41 and 47; Nos. 87 and 89) are counted separately. Region is the country in which the study was conducted. Sample size is the number of participants with prediabetes per study; Nos. 8 and 27 reported only a combined total for mixed populations and are excluded from the median (Category 2, 27 of 29 studies; all studies, 46 of 48). CGM system is the manufacturer of the device named in **Table 2**. Wear duration is the longest continuous monitoring period per assessment (upper bound where a range was given). Intervention or follow-up duration is the longest intervention or follow-up period per study condition. RCT, randomized controlled trial. This table is descriptive only and no quantitative synthesis was attempted.

## 3 Results

Study selection is summarized in **Figure 1** (PRISMA-ScR flow diagram). The supplementary database search and the final composition of the included set are also shown in **Figure 1**. Characteristics of the 48 studies with extracted data are summarized in **Table 2**. **Table 1** provides a descriptive quantitative summary (counts and percentages) of the included studies by synthesis category. Findings are synthesized narratively below (Sections 3.1-3.5), organized by the five categories specified in the registered protocol.

**Table 2:** Characteristics of included studies (n=48).

No.	Author (Year)	Country	Design	PreDM n	CGM device	Wear duration	Cat.
2	Ko (2025) (41)	South Korea	Prospective single-arm cohort	73	FreeStyle Libre 1	2 wk	2
4	Rodriguez (2026) (22)	USA	Cross-sectional (AI-READI)	319	Dexcom G6	≤10 d	1
5	Rama Chandran (2025) (18)	Singapore	Cross-sectional	27	FreeStyle Libre Pro	≤14 d	1
7	Yost (2020) (30)	USA	Single-arm pilot (mixed methods)	15	FreeStyle Libre (blinded to personal)	approx. 20–22 d	2
8	Park (2026) (44)	South Korea	Prospective observational	NR (mixed, total n=110)	FreeStyle Libre	approx. 2 wk	2
9	Rizos (2024) (21)	Greece	Matched-pair observational	18	Envision Pro	7 d	1
10	Cichosz (2025) (14)	Denmark	Cross-sectional (5 pooled cohorts)	133	Mixed (5 studies)	2–7 d	1
11	Koide (2025) (38)	Japan	Sequential open-label	23	FreeStyle Libre	14 d	2
15	Hall (2018) (20)	USA	Observational cohort	14	Dexcom G4	2–4 wk	1
16	Luo (2022) (28)	China	3-arm RCT	253	FreeStyle Libre	14 d × 2	2
19	Spartano (2025) (12)	USA	Cross-sectional (Framingham)	463	Dexcom G6 Pro	7–10 d	1
20	Bakhshi (2025) (13)	USA	Cross-sectional	292	Dexcom G6 Pro	10 d	1
24	Kaufman (2023) (55)	India	Cross-sectional (2 cohorts)	98	FreeStyle Libre Pro	14 d	3
26	Veluvali (2025) (45)	USA	Retrospective cohort	98	FreeStyle Libre 2	14 d	2
27	Barua (2025) (49)	USA	RCT secondary analysis	NR (mixed, total n=76)	FreeStyle Libre Pro	≤14 d	2
28	Richardson (2024) (42)	USA	Prospective pilot	27	Dexcom G6	10 d × 2	2
38	Torii-Goto (2025) (43)	Japan	Prospective controlled	18	FreeStyle Libre Pro	14 d × 2	2
39	de Bie (2023) (53)	Netherlands	RCT	51	FreeStyle Libre	10 d × 2	2
40	Farahmand (2025) (26)	USA	ML model development (AI-READI)	207	Dexcom G6	10 d	1
41	Færch (2021) (56)	Denmark	RCT *post-hoc* analysis	120	iPro2	6 d × 3	3
46	Luk (2018) (54)	Hong Kong	RCT	60	iPro2	72 h × 3	2
47	Færch (2021) (52)	Denmark	4-arm RCT	120	iPro2	6 d × 4	2
48	Hibi (2019) (33)	Japan	Crossover RCT	9	Medtronic CGMS	approx. 1.5 d	2
51	Bonikowske (2021) (59)	USA	Pilot crossover	10	Medtronic iPro2	1 wk × 2	5
53	van Dijk (2012) (39)	Netherlands	Crossover	15	GlucoDay S	3 d × 3	2
54	Kordowski (2022) (32)	Germany	Single-arm, open-label (real-life)	62	FreeStyle Libre	14 d × 2	2
55	Chen (2020) (29)	China	RCT	100	FreeStyle Libre	14 d × 2	2
58	Santos-Báez (2025) (23)	USA	Prospective pilot	36	FreeStyle Libre Pro	14 d	1
59	Bruno (2024) (36)	USA	Crossover	10	NR	6 d × 2	2
61	Zaman (2024) (50)	USA	RCT secondary analysis	7 (subgroup)	FreeStyle Libre Pro	7 d × 2	2
62	Chong (2026) (25)	Malaysia	Observational cohort	120	NR	7 d	1
63	Kloura (2026) (37)	Denmark	RCT secondary analysis	46	iPro2	7 d × 3	2
64	Abraham (2026) (40)	USA	Crossover	6 (mixed cohort)	Dexcom G6	3 d × 4	2
66	Dimova (2025) (17)	Bulgaria	Cross-sectional	42	FreeStyle Libre Pro	14 d	1
70	Bañuls (2016) (31)	Spain	RCT	44	Dexcom SEVEN Plus	3 d × 2	2
71	Parr (2018) (34)	Australia	Crossover	13	iPro2	approx. 1.5 d × 2	2
78	Chen (2013) (15)	China	Cross-sectional	52	Medtronic MiniMed	72 h	1
81	Shilo (2024) (57)	Israel	Prospective cohort (10K project)	2,718 (baseline); n=8,315 total cohort	NR	approx. 7 d (mean 7.16 ± 3.17 d)	3
82	Diaz-Rizzolo (2025) (35)	USA	Observational	33	FreeStyle Libre Pro	14 d	2
83	Dimova (2022) (16)	Bulgaria	Cross-sectional	59	FreeStyle Libre Pro	24 h	1
86	Ma (2025) (48)	China	RCT	41	Si Sensor	14 d × 2	2
87	Basiri (2024) (46)	USA	RCT	30	NR	10 d × 3	2
89	Basiri (2025) (47)	USA	RCT (companion of No.87)	30	NR	10 d × 3	2
90	Gravesteijn (2023) (61)	Netherlands	RCT crossover	34	FreeStyle Libre Pro	48 h	2
91	Peh (2020) (62)	Singapore	RCT crossover (non-blind)	13	Medtronic MiniMed iPro2	27 h	2
92	Dimova (2023) (63)	Bulgaria	Cross-sectional	53	FreeStyle Libre Pro	14 d	1
93	Gulati (2023) (64)	India	RCT crossover (2-phase)	60	Medtronic MiniMed iPro2	3 d × 2	2
94	Carletti (2025) (65)	USA	Prospective cohort (cross-sectional CGM analysis; external validation cohort)	79 (of 347)	Dexcom G6 (FreeStyle Libre Pro in validation cohort)	10 d	1

Cat, narrative synthesis category (1, CGM metrics characterization in prediabetes; 2, CGM-guided interventions; 3, CGM for detection/screening; 4, CGM and diabetes progression [no studies]; 5, CGM and metabolic/complication risk). PreDM n, prediabetes-specific sample size as reported (or total n with a note where not separately reported for a mixed cohort). Rows ordered by study number (No.), matching **Supplementary Table S4**. NR, not reported in the accessible full text.

10 additional records met eligibility criteria based on title and abstract but had no freely accessible full text and could not be obtained without direct purchase or a reprint request to the authors. Because eligibility could not be fully confirmed and data could not be extracted, these records were excluded from the final included set; see Methods, Section 2.4, and Discussion, Limitations. Five further studies were added after the original PubMed-based screening pipeline: four (**Table 2**, Nos. 90–92 and 94) were identified through the supplementary Europe PMC/Scopus cross-database check described in Methods, Section 2.3, and Discussion, Section 4.5, and one (No. 93) was reinstated after *post hoc* re-screening of AI-excluded records; their data were extracted using the same standardized form, yielding a total of 49 included studies. In addition, on further verification of the source article (51), No. 60 (Jospe et al.) was found to rely predominantly on self-monitored blood glucose (glucometer), with CGM as an undifferentiated, unquantified optional device choice and no CGM-specific outcome data reported; this study could not be confirmed to meet this review’s Concept eligibility criterion (rtCGM/isCGM use) and has been excluded, yielding a final total of 48 included studies.

### 3.1 CGM metrics characterization in prediabetes (15 studies)

A substantial body of evidence has used CGM to describe how glycemic patterns differ across the spectrum from normoglycemia to prediabetes to overt diabetes, independent of any intervention. In a large community-based Framingham cohort (n=1175), Spartano et al. (12) showed that individuals with prediabetes spent a mean of 96.1% of time in the conventional 70–180 mg/dL range, but only 76.9% in the tighter 70–140 mg/dL range, with prediabetic participants already spending roughly 19% of time above 140 mg/dL and 3.3% above 180 mg/dL. These figures rose sharply in participants with diabetes (72.1% and 27.4% of time in 70–180 and >180 mg/dL, respectively).

This progressive worsening across glycemic status was echoed in two large cross-sectional cohorts. Bakhshi et al. (13) (n=677) found progressively higher mean glucose, coefficient of variation (CV), mean amplitude of glycemic excursions (MAGE), J-index, and time >140 mg/dL from normoglycemia to prediabetes. Cichosz et al. (14) (pooled n=836 across five cohorts) reported that time below range, time above 140 mg/dL, and several novel indices (mobility, MCI, glycemic risk index) already differed significantly between normoglycemic and prediabetic groups, whereas SD and CV did not. This suggests that conventional variability metrics may be less sensitive than newer or threshold-based indices for detecting early dysglycemia.

Chen et al. (15) (n=220) demonstrated that glycemic variability (GV) indices, including standard deviation (SD), mean amplitude of glycemic excursions (MAGE), continuous overall net glycemic action (CONGA1), mean of daily differences (MODD), and high blood glucose index (HBGI), rose progressively from normal glucose tolerance to isolated IGT to diabetes, and that this rise correlated strongly and inversely with the oral disposition index and other measures of insulin secretion and sensitivity (r = -0.37 to -0.72), indicating that increased glycemic variability in prediabetes is mechanistically linked to declining beta-cell compensation rather than being an artifact of monitoring. Two studies from the same Bulgarian research group examined this relationship further. Dimova et al. (16) (n=104) reported a similar progressive rise in GV indices across the same glycemic continuum. Their companion study showed that CGM-derived variability differed by degree of OGTT abnormality, comparing isolated 1-hour hyperglycemia with isolated IGT, and that results were broadly consistent whether CGM was worn under free-living or standardized 24-hour conditions, supporting the robustness of CGM-derived GV as a physiological marker across monitoring contexts (17).

Establishing reference ranges in a non-Western population, Rama Chandran et al. (18) (n=151, Singapore, predominantly Chinese ethnicity) found that normoglycemic individuals spent a median of 97.3% of time in a 54–140 mg/dL tight range, and that among all CGM indices tested, time above 140 mg/dL (TAR140) had the best discriminative ability for prediabetes (AUROC 0.72, sensitivity 48%, specificity 88% at a 5.64% threshold), while CV did not differ significantly between normoglycemic and prediabetic groups. Notably, the prediabetic group had a lower median BMI (22.8 kg/m²) than is typically reported in Western cohorts, consistent with a “thin-fat” Asian phenotype in which dysglycemia can occur at comparatively low adiposity (19). This finding has implications for how prediabetes risk is assessed across ethnic groups.

At the individual level, Hall et al.’s influential “glucotype” study used unsupervised clustering of CGM waveforms to show that even individuals classified as normoglycemic by standard testing spent a meaningful proportion of time (approximately 15% and 2%) in prediabetic and diabetic glucose ranges, respectively (20). Roughly a quarter of nominally “normal” participants reached prediabetic or diabetic glucose excursions after standardized meal challenges, highlighting substantial glycemic heterogeneity that oral testing alone does not capture. Consistent with this theme, Rizos et al. (21) found that in a matched-pair comparison, prediabetic individuals had significantly higher mean glucose, higher glycemic variability (CV, MAGE), and greater post-OGTT AUC than normoglycemic controls despite similar demographic profiles.

Several studies examined how demographic, dietary, or clinical factors modulate CGM metrics within the prediabetic population itself. Rodriguez et al. (22) (n=972) reported that the association between HbA1c and CGM indices, including mean glucose, time in range (TIR), time in tight range (TITR), SD, MAGE, MODD, and the 24-hour continuous overall net glycemic action (CONGA), was statistically significant in prediabetes but substantially attenuated compared with type 2 diabetes, raising caution about extrapolating HbA1c-CGM relationships validated in diabetes to the prediabetic range. Santos-Báez et al. (23) found that fasting glucose and habitual MAGE were the strongest predictors of both OGTT- and meal-related postprandial glucose response in prediabetes and early type 2 diabetes, whereas age, BMI, and habitual sleep and activity were not. This echoes earlier large-cohort work showing high inter-individual variability in postprandial glycemic response to identical meals (24). Chong et al. (25) linked evening chronotype to higher 24-hour mean CGM glucose and shorter sleep duration to lower TIR in a Malaysian prediabetes cohort, implicating circadian and sleep factors in glycemic control independent of classical risk factors. Finally, Farahmand et al. (26) demonstrated that a multimodal Transformer-based deep-learning model (AttenGluco) incorporating CGM and activity data, built on the AI-READI dataset (27) used by Rodriguez et al. above, improved long-horizon glucose forecasting accuracy specifically in a prediabetes cohort compared with baseline models. This illustrates an emerging application of CGM data beyond simple descriptive metrics.

Taken together, these 15 studies show that CGM consistently reveals a graded, physiologically coherent worsening of glycemic control and variability from normoglycemia through prediabetes to diabetes. This pattern is often present even when conventional single time-point tests (HbA1c, FPG) appear only mildly abnormal or unchanged. However, which specific metrics best capture this gradient is inconsistent across studies, with some favoring MAGE or CV and others favoring threshold-based time-in-range indices. In addition, the HbA1c-CGM relationship itself appears weaker in prediabetes than in diabetes. This is an important limitation to note when using CGM as a surrogate for HbA1c in this population. A supplementary cross-database search identified two further characterization studies. Dimova et al. (63) found glycemic variability (measured via 14-day FreeStyle Libre Pro CGM) to be higher in impaired glucose tolerance (n=53) than normal glucose tolerance (n=41), and to worsen with greater carbohydrate and refined-grain intake, consistent with the graded dysglycemia pattern described above. Carletti et al. (65), in a US cohort that included 79 adults with prediabetes, reported that CGM-derived glucose-spike metrics in prediabetes were closer to those of normoglycemia than to those of type 2 diabetes, with substantial heterogeneity within each group.

### 3.2 CGM-guided interventions (29 studies)

This was by far the largest category, encompassing dietary, meal-timing, exercise, pharmacological/supplement, and digital-coaching interventions in which CGM was used either as an outcome measure, a feedback tool, or both.

#### 3.2.1 Dietary composition and quality

Several RCTs compared specific dietary patterns. Luo et al. (28) (n=253) found that 6 months of isocaloric-restricted Mediterranean, traditional Chinese, or control diets produced similar weight loss (5.05-5.72 kg) with no between-group differences in fasting glucose, insulin, or the OGTT-derived Matsuda index. However, the control (low-plant) diet was associated with significantly more CGM-measured hypoglycemic time than the Mediterranean diet, suggesting that diet quality, not calorie restriction alone, modulates glycemic stability. Chen et al. (29) (n=100) tested low-carbohydrate dietary coaching delivered via a companion mobile app and found a CGM-verified reduction in postprandial hyperglycemic duration (3.27 to 2.34 h/day), and Yost et al. (30) also reported a similar trend in a comparable low-carbohydrate coaching pilot, though not statistically significant. Bañuls et al. (31) tested an inositol-enriched beverage and found a significant reduction in overnight glucose. Kordowski et al. (32) tested a synbiotic supplement and found improvements in postprandial glucose (iAUC), fasting glucose, and HbA1c, with effects concentrated in a prediabetic rather than normoglycemic subgroup; neither study was adequately powered for definitive conclusions. Two further dietary studies identified through the supplementary cross-database search examined premeal or regular almond consumption using CGM as an outcome measure. Gulati et al. (64) (n=60, Asian Indians with prediabetes) found that 20 g of almonds consumed 30 minutes before each major meal reduced postprandial hyperglycemia (10.1% reduction in CGM-derived glucose AUC) over a 3-day CGMS monitoring period. In contrast, Gravesteijn et al. (61) (n=34 completers, prediabetes) found that 50 g of daily almonds over 16 weeks did not improve, and in some respects slightly worsened, 48-hour CGM-derived glucose measures (higher minimum glucose, P = 0.038) alongside a significant decrease in whole-body insulin sensitivity (P = 0.002) on euglycemic clamp, illustrating that CGM-detectable effects of dietary interventions in this category are not uniformly favorable.

#### 3.2.2 Meal timing, frequency, and time-restricted eating

Hibi et al. (33) showed in a crossover design that dividing identical caloric intake into 9 vs 3 meals per day significantly reduced daytime and nighttime maximum glucose and glucose range in men with impaired fasting glucose. Parr et al. (34) found that a high- versus low-energy breakfast altered the distribution of postprandial glucose exposure across the day, with more exposure after breakfast and less after lunch, without changing 24-hour mean glucose or variability. Diaz-Rizzolo et al. (35) examined how the timing and composition of the last evening meal predicted next-morning fasting glucose via CGM-derived overnight metrics. Two time-restricted eating (TRE) trials produced more clearly positive signals. Bruno et al. (36) (n=10, 6-day crossover) found that an early 8-hour eating window reduced MAGE by 17% and CGM time in mild hyperglycemia (140–180 mg/dL) by 52% without increasing nocturnal hypoglycemia. Kloura et al. (37) (RESET trial secondary analysis, n=46) found a significant but transient reduction in nocturnal mean glucose at 6 weeks with 10-hour TRE that was no longer present at 3 months, with no between-group effect on CV or TIR, which was already near-ceiling at baseline. A further meal-timing study identified through the supplementary cross-database search, Peh et al. (62) (n=13, pre-diabetic Asian males), used 27-hour CGM (Medtronic MiniMed iPro2) to show that consuming a high-glycemic-index breakfast significantly reduced the postprandial glucose, insulin, and non-esterified fatty-acid response at the subsequent lunch meal (P = 0.001) compared with skipping breakfast, consistent with a protective “second-meal effect” of breakfast consumption.

#### 3.2.3 Exercise

Koide et al. (38) showed that a single 30-minute post-lunch walk significantly reduced 1-hour postprandial AUC and improved percent time in tight range in sedentary Japanese office workers with mildly elevated HbA1c. Van Dijk et al. (39) found that a single bout of either resistance- or endurance-type exercise reduced subsequent 24-hour hyperglycemic duration similarly in participants with IGT and type 2 diabetes, with a small, non-significant increase in hypoglycemia. Abraham et al. (40) (n=11, mixed prediabetes/T2D) found that 7 days of moderate exercise lowered mean 24-hour glucose, while evening red wine consumption independently reduced the evening postprandial glucose response, with no interaction between the two exposures.

#### 3.2.4 CGM-integrated behavioral and digital coaching programs

The largest subgroup of intervention studies combined CGM feedback with structured education, digital coaching, or app-based behavior change, generally in the context of diabetes prevention program (DPP)-style curricula. Richardson et al. (42) established the feasibility and acceptability of adding CGM to a National DPP lifestyle program, and Ko et al. (41) reported the first workplace-based implementation of a CGM program with structured education. Richardson’s pilot specifically reported high opt-in (77%) and acceptability (90%), along with a baseline CGM-measured TIR of approximately 97%. However, this pilot was not powered for pre/post comparison. Torii-Goto et al. (43) found that 2 months of isCGM plus lifestyle coaching significantly improved TIR in a prediabetic subgroup but not in a normoglycemic control group receiving identical coaching, while HbA1c did not change in either group. This shows that short-term improvement visible on CGM does not necessarily translate into longer-term change in glycemic biomarkers like HbA1c. Park et al. (44) used AI-supported digital coaching with CGM feedback and found the largest HbA1c reductions in participants with the highest baseline glycemic variability. Veluvali et al. (45) similarly found the largest TIR improvements in users with the lowest baseline TIR. Both patterns suggest a ceiling effect in metabolically healthier participants. Basiri and colleagues’ companion RCT (46, 47) (n=30, same cohort across both papers) found that adding real-time CGM feedback to individualized nutrition therapy significantly improved TIR (from 95.1% to 97.9%), the glucose management indicator (GMI), and %CV relative to a masked-CGM control arm. These glycemic gains were accompanied by significant improvements in whole-grain and plant-protein intake and sleep efficiency in the feedback arm. In the only long-term (2-year) trial in this category, Ma et al. (48) (n=41) found that adding real-time CGM feedback to individualized diabetes education produced significantly lower HbA1c than education alone at both 1 year (5.68% vs. 6.36%, p=0.007) and 2 years (5.74% vs. 6.1%, p=0.033), despite no between-group difference in weight change. This raises the possibility of a CGM-specific behavioral mechanism independent of weight loss. By contrast, two secondary analyses of behavioral weight-loss RCTs reached a different conclusion. Barua et al. (49) found that CGM-measured glycemic improvement, particularly daytime TIR, tracked with the degree of weight loss achieved rather than with the specific intervention arm. Zaman et al. (50) (n=44) compared early time-restricted eating plus caloric restriction against caloric restriction alone and similarly found no between-group differences in CGM-derived glucose metrics once weight change was accounted for, reinforcing that the degree of weight loss, rather than intervention type, drove glycemic improvement.

#### 3.2.5 Pharmacological and supplement interventions

Færch et al. (52) reported that in a 4-arm PRE-D trial (n=120), 13 weeks of dapagliflozin produced the largest CGM-measured reduction in MAGE (-17.1% vs control) among dapagliflozin, metformin, exercise, and control arms. Exercise showed a similar but non-significant trend, and metformin showed no effect on variability. HbA1c changes were minimal and transient across all arms. Two supplement trials were essentially null. De Bie et al. (53) (n=51) found that oral GABA supplementation did not improve OGTT postprandial response or any CGM-derived variability metric versus placebo. Luk et al. (54) found that a galactomannan-based fiber supplement (BTI320) did not significantly change HbA1c or fructosamine, though the low-dose arm showed CGM-detected reductions in postprandial glucose AUC without a clear dose-response relationship.

Taken together, across this heterogeneous group of studies, CGM-detectable improvements in glycemic variability or time-in-range were achievable through diverse routes. These included dietary quality, meal timing and frequency, time-restricted eating, single bouts of exercise, SGLT2-inhibitor therapy, and structured digital coaching. However, these CGM-level changes did not consistently translate into significant HbA1c or fasting plasma glucose improvement over the typically short trial durations. A notable exception was the one 2-year trial by Ma et al., along with several diet-quality trials that were adequately powered and long enough to detect biomarker change.

Several studies reported time in range, time above range, and time below range using non-standard thresholds rather than the standard 70–180 mg/dL definition. Barua et al. and Basiri and colleagues used a narrower “tight range” of 70–140 mg/dL alongside the standard definition. Bruno et al. additionally reported a separate mild-hyperglycemia band of 140–180 mg/dL. This inconsistency complicates cross-study comparison (see Discussion, Section 4.4).

### 3.3 CGM for detection/screening (3 studies)

Two of the three included studies in this category directly evaluated CGM’s potential as a prediabetes screening or diagnostic tool using traditional validation approaches. Kaufman et al. (55) (n=384, pooled across calibration and validation cohorts) developed and validated a novel CGM-derived classification framework called FLAG (functional assessment of glucose homeostasis), based on a proportional-integral homeostatic model. This framework achieved a sensitivity of 86% for prediabetes and 100% for diabetes, comparable to HbA1c and substantially better than OGTT, which had only 18-24% sensitivity for prediabetes. The authors proposed CGM as a pre-screening tool ahead of confirmatory testing.

In a *post-hoc* Bland-Altman analysis of the PRE-D trial, Færch et al. (56) examined 306 paired measurements from 120 participants. They found that professional CGM (iPro2) systematically underestimated venous OGTT glucose by 4-13% at most timepoints. This proportional bias worsened at higher glucose concentrations. These findings indicate that CGM cannot currently substitute directly for OGTT-based diagnosis, though it may be useful as a complementary or pre-screening signal.

A third, considerably larger study, published in Nature Medicine, substantially strengthens the case for further investigation in this area. Shilo et al. (57) studied a large nondiabetic Israeli cohort of 8,315 individuals. They used repeated CGM-derived morning fasting glucose measurements, taken over a mean of 7.16 ± 3.17 days per participant, to quantify day-to-day intrapersonal fasting glucose variability and explore its diagnostic implications. The standard deviation of this intrapersonal variability was 7.52 ± 4.31 mg/dL. Strikingly, of 5,328 individuals classified as having normal fasting glucose based on a single baseline measurement, only 57% remained classified as normal across all subsequent measurements: 40% were reclassified into the prediabetes range, and a further 3% into the suspected or diagnosed diabetes range, based on repeated CGM-derived measurements alone. This large-scale demonstration that single-timepoint fasting glucose measurement resulted in different glycemic classification when repeated CGM-derived measurements were considered is, to our knowledge, the strongest evidence in this review’s included literature that CGM could meaningfully improve the accuracy of prediabetes identification, and reframes this category as an area of genuine emerging evidence rather than pure evidence gap.

Taken together, the evidence base for CGM as a screening or diagnostic tool in prediabetes comprises three studies with somewhat divergent but complementary implications. Færch et al. found that CGM disagrees with venous OGTT glucose by a clinically meaningful margin, which argues against directly substituting CGM for existing diagnostic tests. However, two other findings point in a different direction. Kaufman et al. validated a classification model, and Shilo et al., in a much larger cohort, demonstrated that single-timepoint fasting glucose measurement resulted in different classification when repeated CGM-derived measurements were considered. Together, these suggest that CGM’s value may lie less in replacing OGTT or HbA1c as a diagnostic threshold test, and more in capturing the day-to-day variability that a single blood draw cannot. This remains an area warranting substantially more research, including prospective validation of CGM-based reclassification against hard clinical outcomes.

### 3.4 CGM and diabetes progression (0 studies)

No study among the 48 included studies used CGM-derived metrics as a predictor of, or outcome related to, longitudinal progression from prediabetes to overt type 2 diabetes. Several intervention studies followed participants for up to 6 months (Luo et al., Barua et al., Kloura et al.), and one followed participants for 2 years (Ma et al.). However, none of these studies reported diabetes conversion as an outcome. This absence is itself a notable finding of this review. No eligible study addressed CGM-based prediction of progression to type 2 diabetes, which we highlight as a priority direction for future longitudinal research. This gap can be considered alongside the related work by Black et al., already discussed in the Introduction. Notably, CGM-based progression prediction has already been demonstrated outside this review’s scope, in a study of autoantibody-positive children progressing to type 1 diabetes (58). This shows that the methodology itself is feasible, but it has simply not yet been applied to adult prediabetes or type 2 diabetes progression in the population addressed by this review.

### 3.5 CGM and metabolic/complication risk (1 study)

Only one included study focused primarily on CGM in relation to broader metabolic or complication risk, rather than on glycemic control itself. Bonikowske et al. (59) conducted a small pilot study of 10 women with impaired fasting glucose. They found that sedentary time was a significant independent predictor of CGM-measured blood glucose, after adjusting for diet and physical activity. Participants wore CGM for one week in a seated-desk condition and, following a 4-week acclimation period, for one week in a sit-stand condition. Alternating sitting and standing at a height-adjustable desk did not significantly alter overall glucose levels compared with the seated condition. However, of the 27 eligible participants, 17 did not complete the study, including 5 who experienced CGM device malfunction, and glucose was measured for only one week per condition. Both the substantial attrition and the short measurement window likely limited the study’s ability to detect a modest effect.

As with the previous category, the evidence here is minimal, comprising just one study, and does not permit generalizable conclusions about CGM’s utility for capturing broader cardiometabolic or complication risk in prediabetes. This should also be flagged as a research gap.

## 4 Discussion

### 4.1 Principal findings

This scoping review mapped 48 studies, all with extracted data, examining the use of continuous glucose monitoring (CGM) in adults with prediabetes, organized into five pre-specified categories. Three findings stand out.

First, glycemic control and variability worsened in a stepwise fashion from normoglycemia through prediabetes to diabetes, a pattern that was consistent across the characterization studies in this review.

Second, interventions across several categories, such as diet, meal timing, exercise, medication, and digital coaching, could all shift CGM metrics in a favorable direction. However, these shifts rarely carried through to standard biomarkers such as HbA1c.

Third, and perhaps most important for setting a future research agenda, this review identified two substantial evidence gaps and one emerging but still limited area. The first gap is that no study used CGM to predict or characterize progression from prediabetes to overt type 2 diabetes. The second gap is that only one study addressed CGM in relation to broader metabolic or complication risk beyond glycemic control itself. The emerging area is the use of CGM as a prediabetes screening or diagnostic tool: three studies addressed this, including one large cohort study (n=8,315) showing that single-timepoint fasting glucose measurement resulted in different glycemic classification when repeated CGM-derived measurements were considered, though this area still warrants considerably more prospective validation work.

### 4.2 CGM as a more sensitive window into early dysglycemia

The characterization studies included here converge on a consistent picture. Prediabetes is not a single, static state but a period of substantial and measurable glycemic heterogeneity that intermittent testing, including HbA1c, fasting glucose, and even the 2-hour OGTT value, does not fully capture. The “glucotype” concept illustrates this point well. A meaningful minority of individuals classified as normoglycemic by standard testing nonetheless show prediabetic- or diabetic-range glucose excursions on CGM. Together, these findings suggest that current diagnostic thresholds, calibrated to single-timepoint tests, may not fully reflect the underlying continuum of glycemic dysregulation.

At the same time, this review found direct evidence for caution. The HbA1c-CGM relationship itself is weaker in prediabetes than in diabetes. Professional CGM can also systematically disagree with OGTT-based venous glucose by a clinically meaningful margin. Together, these findings argue against simply substituting CGM for existing diagnostic tools without further validation work.

### 4.3 Interventions: CGM-visible change without consistent biomarker change

Interventions also differed in how CGM itself was used: in some studies, CGM data were shown to participants in real time as direct feedback intended to guide behavior change, whereas in others CGM was blinded and used primarily to measure the glycemic effects of a non-CGM intervention (e.g., a dietary, pharmacological, or exercise protocol). This distinction should be interpreted with some caution: even when CGM data were not visible to participants, the act of wearing a monitoring device may itself have influenced self-management behavior (a Hawthorne-type effect), so blinded CGM use cannot be assumed to be behaviorally inert. Nonetheless, studies that provided direct real-time feedback tended to show the largest effects in participants who started with the poorest glycemic control, consistent with a ceiling effect. A striking pattern across the 29 intervention studies in this review is a frequent dissociation. CGM-detected improvement, whether in time-in-range, glycemic variability, or postprandial glucose excursions, often did not correspond to improvement in conventional biomarkers such as HbA1c or fasting glucose over the same trial period.

Several explanations seem plausible. First, most trials were short, typically 3 months or less. This is likely insufficient time for CGM-level glycemic improvements to produce a measurable HbA1c change, since the HbA1c assay reflects an approximately 3-month erythrocyte lifespan. Second, several studies enrolled participants with near-ceiling baseline glycemic control (Kloura et al., baseline TIR of 99.5%; Richardson et al., baseline TIR of 96.6-98.7%). This left little room for detectable improvement in either CGM metrics or HbA1c. Third, as noted above, feedback-based and blinded-measurement designs were sometimes conflated within comparisons.

Only one included trial followed participants for as long as 2 years, and it was the only trial to report a durable between-group HbA1c difference. This pattern is consistent with the hypothesis that trial duration, rather than intervention type, may be the binding constraint on detecting biomarker-level change.

### 4.4 Methodological heterogeneity as a barrier to synthesis

A consistent challenge in synthesizing this literature was inconsistency in how CGM-derived metrics were defined and reported. Time-in-range was reported using the standard consensus 70–180 mg/dL threshold in most studies. However, several studies used a narrower “tight range” instead: Basiri et al. (46, 47) used 70–140 mg/dL, while Rama Chandran et al. (18), Barua et al. (49), and Zaman et al. (50) used 54–140 mg/dL. None of these studies provided a clear justification for the specific threshold chosen. This variability meaningfully complicates cross-study comparison and, in the view of this review, represents a more fundamental barrier to evidence synthesis in this field than any single study’s individual limitations. We would encourage future prediabetes-focused CGM research to report time-in-range using the internationally standardized 70–180 mg/dL threshold (7) as a common reference point, even when a study’s primary analysis uses an alternative threshold for population-specific reasons.

### 4.5 Strengths and limitations

Several aspects of this review’s methodology support the accuracy and reliability of the synthesis presented here. The protocol was prospectively registered on OSF, and reporting followed PRISMA-ScR standards (11). Eligibility screening of the 89 candidate records was conducted independently by two reviewers, using full text where needed to resolve ambiguous cases, and data extraction was systematic and PMID-verified. Missing data items were explicitly coded as “not reported” rather than imputed. Together, these steps reduced the risk of extraction error or unsupported inference.

Several limitations should be considered when interpreting this review. First, only a single database (PubMed) was searched; while PubMed has broad coverage of the biomedical literature relevant to this topic, some eligible studies indexed only in other databases (e.g., Embase, Web of Science) may have been missed, and this single-database approach is the most significant methodological limitation of this review. To partially address this, we conducted supplementary searches of Europe PMC and Scopus on October 2, 2026, using Boolean queries adapted to each database’s field-tag syntax (Europe PMC: TITLE_ABS; Scopus: unscoped title/abstract/keyword matching) while preserving the same concept terms as the registered PubMed string (full query text provided in **Supplementary File 1**: Multi-Database Literature Search Documentation; record-level results in **Supplementary Table S1**). The Europe PMC search (470 records) identified no peer-reviewed, eligible study missed by the PubMed-only search strategy: of 423 MEDLINE-indexed records, 388 overlapped with our 436-record PubMed export and the remaining 35 were records indexed after our original search date; the remaining 47 non-MEDLINE-indexed records (37 preprints, 9 conference abstracts, 1 other) would not have met this review’s EC3 primary-research-report criterion. The Scopus search (772 records, broader due to indexed-keyword matching and requiring multi-stage filtering) identified 4 eligible studies not captured by the PubMed search: Gravesteijn et al. (61), Peh et al. (62), Dimova et al. (63), and Carletti et al. (65). The records excluded by the AI-assisted title/abstract screening of this supplementary search were likewise not individually verified by human reviewers; a random sample of 100 of these 221 records (fixed random seed) was therefore independently re-screened on title (with the linked record opened where needed) by both authors (H.J.L. and J.K.K.), and all 100 were confirmed as exclusions (0/100 false negatives; exact 95% confidence interval 0% to 3.6%). A further study, Gulati et al. (64), was present in the PubMed export but had been excluded in the AI-assisted first pass, and was reinstated after the *post hoc* re-screening described below. These 5 studies were data-extracted using the same standardized form and added to the included set (**Table 2**, Nos. 90-94), yielding a total of 49 included studies at that point, as summarized in **Figure 1**. One further study originally in the included set, Jospe et al. (51) (No. 60), was subsequently excluded on closer verification of its reliance on self-monitored blood glucose rather than confirmed CGM use, yielding a final total of 48 included studies. This supplementary check therefore both demonstrates concretely why single-database (PubMed-only) coverage is a genuine limitation of the original search, as acknowledged above, and shows that this limitation was identified and partly remedied during the review process. Second, of the 59 records provisionally included after title/abstract and, where needed, full-text screening, free full-text access (via PubMed Central [PMC] or the publisher’s own open-access site) could be confirmed for 49 (83.1%), of which 44 were included after the exclusion of 2 records under EC5 and 3 records available only in Chinese-language full text; because access was obtained through these open-access routes rather than institutional login credentials, access to these studies was not dependent on institutional subscription. The remaining 10 records (16.9%) had no free full-text link available and were not accessible without direct purchase or a reprint request to the authors; as eligibility could not be fully confirmed and data could not be extracted for these records, they were excluded from the final included set rather than retained without extracted data, which is acknowledged as a limitation of this review’s reliance on freely accessible full text. Third, screening and eligibility assessment were restricted to English-language full text; 3 otherwise-eligible studies available only in Chinese-language full text were excluded on this basis, which may underrepresent research conducted in non-English-speaking settings, particularly China, where several of this review’s included studies originated. Fourth, as in any scoping review, no formal risk-of-bias or methodological quality appraisal was performed, and the findings summarized here should not be interpreted as a quality-weighted or effect-size-pooled synthesis. Fifth, a minority of included studies enrolled mixed prediabetes/type 2 diabetes cohorts or cohorts that were predominantly normoglycemic with only a small prediabetes subgroup, without separate prediabetes-specific subgroup analysis in the original publication (40, 44, 49, 50). These studies’ contributions to this review’s synthesis are correspondingly less precise and are flagged individually in the extraction table. Sixth, the initial title/abstract screening pass (436 to 89 records) was conducted by a large language model rather than by human reviewers, given the volume of records, whereas the subsequent eligibility assessment (89 to 59 records), based on title and abstract with full text opened for ambiguous records, was conducted independently by two human reviewers as described in Methods, Section 2.4. The AI-assisted first pass was not independently re-verified in its entirety at the time of the original screening; as a partial check at that time, a small sample of literature adjacent to but not meeting our eligibility criteria, comprising 4 review articles identified through supplementary searches conducted after the original screening, was assessed against EC3 and confirmed to have been appropriately excluded, supporting the specificity of the AI-assisted screening step. We subsequently conducted a *post hoc* human re-screening of a random sample of 100 of the 339 AI-excluded records (fixed random seed for reproducibility), independently re-reviewed by both authors (H.J.L. and J.K.K.) against the same PCC and EC1-EC5 criteria used in the original screening. This audit identified two false-negative exclusions, corresponding to a miss rate of 2% (2/100; exact 95% confidence interval 0.2% to 7.0%): Ben-Yacov et al., 2021, Diabetes Care (PMID 34301736), and Gulati et al., 2023, Eur J Clin Nutr (PMID 36732571). The latter had been excluded by the AI-assisted first pass and, under EC5, by the human re-screen, but on reappraisal used CGM as a clinical outcome measure within a dietary randomized trial, and it was added to the included set (**Table 2**, No. 93). However, as with the 10 included-pipeline records discussed above, free full text of the Ben-Yacov record could not be obtained without institutional subscription or direct purchase; applying the same reports-not-retrieved rule described in Methods, Section 2.4, this record was therefore not added to the included set. A third record, Lutsker et al., 2026, Nature (PMID 41535468), was initially coded as eligible in the reviewers’ re-screen but was reclassified on adjudication as EC5 (validation of a machine-learning foundation model in a mostly non-diabetic cohort, without a defined prediabetes population or clinical glycemic outcome), and was therefore not counted as a false negative. The remaining 239 records in the AI-excluded pool were not individually re-screened, and this remains a residual limitation of the review’s screening methodology.

### 4.6 Research gaps and future directions

This review identified two areas where the evidence base is currently minimal to non-existent, and one area of genuine emerging evidence that nonetheless warrants substantially more research, which we would highlight as priorities for future work.

Most notably, no study in this review used CGM-derived metrics to predict, or as an outcome related to, longitudinal progression from prediabetes to overt type 2 diabetes. This is a notable gap given that such prediction has already been demonstrated as feasible in a related population, namely autoantibody-positive children progressing to type 1 diabetes (58), and given the substantial clinical value that an accurate CGM-based progression risk marker could offer for prediabetes management and diabetes prevention programs (60).

Second, CGM’s relationship to broader cardiometabolic risk and diabetes-related complications beyond glycemic control itself was addressed by only one small pilot study, by Bonikowske et al. (59), leaving this an essentially open question for future longitudinal research.

Third, the potential role of CGM in prediabetes screening or diagnosis was addressed by three studies. One large cohort study (57) (n=8,315) demonstrated that single-timepoint fasting glucose measurement resulted in different glycemic classification when repeated CGM-derived measurements were considered. A smaller classification-model validation study by Kaufman et al. (55) points in the same direction. Together, these two findings suggest real promise for this application. However, a third study (56) in this category found that CGM systematically disagrees with OGTT-based venous glucose by a clinically meaningful margin. This underscores that larger prospective validation studies, directly comparing CGM-derived reclassification against hard clinical outcomes, are still needed before any clinical recommendation can be made. This is consistent with current professional guidance that evidence remains insufficient to support CGM for this purpose at present (6).

### 4.7 Conclusion

CGM offers a more granular, continuous window into glycemic dysregulation in prediabetes than intermittent testing alone, and a wide range of dietary, behavioral, and pharmacological interventions can produce CGM-detectable glycemic improvement in this population. However, several important limitations remain. The translation of these short-term CGM-level changes into durable improvements in conventional glycemic biomarkers is inconsistently demonstrated. Methodological heterogeneity in CGM metric reporting complicates cross-study synthesis. Three clinically important applications of CGM in prediabetes remain substantially underexplored: progression prediction, screening/diagnosis, and complication-risk assessment. Addressing these gaps, alongside greater standardization of CGM-derived outcome reporting, should be priorities for the next generation of prediabetes-focused CGM research.

## Data Availability

The original contributions presented in the study are included in the article/**Supplementary Material**, further inquiries can be directed to the corresponding author/s.
