# Supplementary material for: Continuous glucose monitoring for diabetes prevention in adults with prediabetes: a scoping review
Source: Front Endocrinol (Lausanne). 2026 Oct 28;17:1965925. doi: 10.3389/fendo.2026.1965925 (PMC13752008; doi:10.3389/fendo.2026.1965925)
Supplement: Supplementary file 1 [file SupplementaryFile1.docx]

**Supplementary File 1: Multi-Database Literature Search Documentation**

Continuous Glucose Monitoring for Diabetes Prevention in Adults with Prediabetes: A Scoping Review. Record-level lists for all three databases are provided in Supplementary Table S1 (Supplementary_Table_S1._Search_Records.xlsx). The flow of the supplementary search and the final composition of the included set are shown in Figure 1 of the main manuscript.

**1. Search summary**

| **Database** | **Role** | **Search date** | **Records** | **Eligible studies not captured by PubMed** |
| --- | --- | --- | --- | --- |
| PubMed | Primary (OSF-registered) | 2026-06-29 | 436 | n/a (primary pipeline) |
| Europe PMC | Supplementary cross-check | 2026-10-02 | 470 | 0 |
| Scopus | Supplementary cross-check | 2026-10-02 | 772 | 4 (Table 2, Nos. 90-92 and 94) |

**2. PubMed (primary search)**

| **Search date** | **June 29, 2026 (date of OSF registration)** |
| --- | --- |
| Query string | (prediabetes[MeSH Terms] OR prediabetes[tiab] OR "pre-diabetes"[tiab] OR "impaired glucose tolerance"[tiab] OR "impaired fasting glucose"[tiab] OR "intermediate hyperglycemia"[tiab] OR "dysglycemia"[tiab]) AND ("continuous glucose monitoring"[tiab] OR "continuous glucose monitor"[tiab] OR CGM[tiab] OR "flash glucose monitoring"[tiab] OR "intermittently scanned CGM"[tiab] OR isCGM[tiab] OR FGM[tiab] OR "FreeStyle Libre"[tiab] OR "Dexcom"[tiab]) |
| Filters | None (no date, language, or species restriction) |
| Records | 436 |
| Screening | First pass (AI-assisted): 8 duplicate records removed, 339 records excluded at title/abstract, 89 retained for full-text review. Two authors independently assessed the 89 retained records: 59 provisionally included, 44 after full-text/data-extraction stage. A post hoc random sample of 100 AI-excluded records was independently re-screened by two authors (2 false negatives, 2%): Gulati et al. (PMID 36732571; reinstated, Table 2 No. 93) and Ben-Yacov et al. (PMID 34301736; no free full text, not added). |

**3. Europe PMC (supplementary)**

| **Search date** | **October 2, 2026** |
| --- | --- |
| Query string | (TITLE_ABS:"prediabetes" OR TITLE_ABS:"pre-diabetes" OR TITLE_ABS:"impaired glucose tolerance" OR TITLE_ABS:"impaired fasting glucose" OR TITLE_ABS:"intermediate hyperglycemia" OR TITLE_ABS:"dysglycemia") AND (TITLE_ABS:"continuous glucose monitoring" OR TITLE_ABS:"continuous glucose monitor" OR TITLE_ABS:"CGM" OR TITLE_ABS:"flash glucose monitoring" OR TITLE_ABS:"intermittently scanned CGM" OR TITLE_ABS:"isCGM" OR TITLE_ABS:"FGM" OR TITLE_ABS:"FreeStyle Libre" OR TITLE_ABS:"Dexcom") |
| Records | 470 |

| **Category** | **Records** |
| --- | --- |
| MEDLINE-indexed | 423 |
| in PubMed export (436 records) | 388 |
| not in PubMed export (indexed after the original search date) | 35 |
| Not MEDLINE-indexed (not peer-reviewed primary reports; EC3) | 47 |
| preprints | 37 |
| conference abstracts | 9 |
| other | 1 |
| Eligible studies not captured by PubMed | 0 |

**4. Scopus (supplementary)**

| **Search date** | **October 2, 2026** |
| --- | --- |
| Query string | (prediabetes OR "pre-diabetes" OR "impaired glucose tolerance" OR "impaired fasting glucose" OR "intermediate hyperglycemia" OR dysglycemia) AND ("continuous glucose monitoring" OR "continuous glucose monitor" OR CGM OR "flash glucose monitoring" OR "intermittently scanned CGM" OR isCGM OR FGM OR "FreeStyle Libre" OR Dexcom) |
| Records | 772 |

| **Step** | **Records** |
| --- | --- |
| Records retrieved | 772 |
| Already in the PubMed export (matched by PMID or DOI) | 410 |
| Not in the PubMed export | 362 |
| non-primary document types (review 61, note 18, editorial 14, letter 12, book chapter 10, book 2, erratum 1; EC3) | 118 |
| articles and conference papers | 244 |
| eligible and added (Gravesteijn, Peh, Dimova, Carletti; Table 2, Nos. 90-92 and 94) | 4 |
| not eligible after abstract review (PMID 35142713: no CGM use) | 1 |
| modelling/machine-learning studies (EC5) on title | 6 |
| not adults with prediabetes and CGM on title | 214 |
| full text reviewed, not eligible or outside the search window (reviews 2, healthy or adolescent participants 3, machine-learning/model development 2, prediabetes not analysed separately or CGM not an outcome of interest 2, outside search window 2) | 11 |
| full text reviewed, not included (two reports of one supplementation trial) | 2 |
| excluded on title/abstract review (no free full text obtained) | 6 |

Note: Gulati et al. (PMID 36732571; Table 2, No. 93) was already in the PubMed export (first-pass AI exclusion) and was reinstated after the post hoc re-screening; it is therefore counted among the 410 records already in PubMed, not among the Scopus-only finds.

Note: Records excluded by the AI-assisted title/abstract screening of the Scopus search were not individually verified by human reviewers. The sampling frame was the 221 records excluded on title/abstract (214 not adults with prediabetes and CGM, 6 modelling/machine-learning studies [EC5], 1 without CGM use); a random sample of 100 (fixed random seed 42) was independently re-screened on title by two authors (H.J.L. and J.K.K.), with the linked record opened where needed. All 100 were confirmed as exclusions (0/100 false negatives; exact 95% confidence interval 0% to 3.6%). Record-level results are in Supplementary Table S2 (sheet Scopus_Sample100). The corresponding random sample of 100 AI-excluded PubMed records, re-screened by the same two reviewers, identified 2 false-negative exclusions (2/100; exact 95% confidence interval 0.2% to 7.0%): Gulati et al. (PMID 36732571; reinstated, Table 2, No. 93) and Ben-Yacov et al. (PMID 34301736; not added, no free full text); a third record initially coded eligible (Lutsker et al., PMID 41535468) was reclassified as EC5 on adjudication. These records are in Supplementary Table S2 (sheet PubMed_Sample100).

**5. Reconciliation of the final included count**

| **Step** | **Studies** |
| --- | --- |
| PubMed primary pipeline (after full-text/data-extraction stage) | 44 |
| + Scopus-only finds (Table 2, Nos. 90-92 and 94) | +4 = 48 |
| + Gulati et al. reinstated after post hoc re-screening (No. 93) | +1 = 49 |
| - Jospe et al. excluded on re-verification of the source article (CGM use not confirmed) | -1 = 48 |
| Final included | 48 |
